# Production of dengue virus-like particles serotype-3 in silkworm larvae and their ability to elicit a humoral immune response in mice

**DOI:** 10.1186/s13568-020-01087-3

**Published:** 2020-08-17

**Authors:** Doddy Irawan Setyo Utomo, Sabar Pambudi, Fithriyah Sjatha, Tatsuya Kato, Enoch Y. Park

**Affiliations:** 1 Laboratory of Biotechnology, Graduate School of Science and Technology, Shizuoka University, 836 Ohya, Suruga-ku, Shizuoka, 422-8529 Japan; 2 Center of Pharmaceutical and Medical Technology, Agency for the Assessment and Application of Technology (BPPT), Jl. Kawasan Puspiptek, Gedung I LAPTIAB, Kota Tangerang Selatan, Banten 15314 Indonesia; 3 Department of Microbiology, Faculty of Medicine, Universitas Indonesia, Jl.Pegangsaan Timur 16, Cikini, Jakarta, 10320 Indonesia; 4 Laboratory of Biotechnology, Research Institute of Green Science and Technology, Shizuoka University, 836 Ohya, Suruga-ku, Shizuoka, 422-8529 Japan

**Keywords:** Dengue virus, Capsid, Premembrane, Envelope, Dengue virus-like particle, Silkworm

## Abstract

To develop monovalent dengue virus-like particle for serotype 3 (DENV-LP/3), we prepared and expressed two structural polyprotein constructs using silkworm and Bm5 cells: DENV-3 Capsid-premembrane-envelope (DENV-3CprME) and premembrane-envelope (DENV-3prME). The expressed PA-tagged 3CprME and 3prME polypeptides were partially purified by PA-tag affinity chromatography and had molecular weights of 85 and 75 kDa, respectively. Expressed proteins were separately verified using the following primary antibodies: the anti-PA tag antibody, DENV premembrane polyclonal antibody, and DENV envelope polyclonal antibody. Transmission electron microscopy revealed that these DENV-3CprME and 3prME formed rough, spherical DENV-LPs (DENV-LP/3CprME and DENV-LP/3prME), respectively, with a diameter of 30–55 nm. The heparin-binding assay demonstrated that these DENV-LPs contained the envelope protein domain III on their surfaces. Both DENV-LPs showed an affinity to sera from human dengue patients and immunized mice. Immunization of mice with DENV-LP/3prME significantly induced the level of antibodies compared with DENV-LP/3CprME. These results indicate that DENV-LP/3prME is suitable as a vaccine candidate compared with DENV-LP/3CprME.

## Introduction

Dengue virus (DENV) is a single-stranded RNA arbovirus that has caused a global epidemic, which is most prominent in tropical and subtropical regions of the Americas, Asia, Africa, and the Pacific Islands. The number of cases of dengue has been registered by the World Health Organization (WHO) since the 1950s, In 2016, the WHO estimated a global case rate of over 3.34 million cases a year, compared with a case rate of < 1000 cases a year in the 1950s. It was estimated that ~ 390 million DENV infections occur annually, while another study suggested that 3.9 billion people in 128 countries were at risk for DENV infection. Healthcare costs associated with dengue fever alone add up to approximately 8.9 billion US dollars per year worldwide. Among dengue cases worldwide, approximately 18% result in hospital admissions, 48% result in outpatient visits, and 34% do not result in any medical visits (Añez et al. 2016; Shepard et al. 2016; Yuan et al. 2020).

DENVs are categorized into four distinct serotypes (DENV-1, DENV-2, DENV-3, and DENV-4), and human infection can present as undifferentiated fever, dengue fever, and dengue hemorrhagic fever (Halsey et al. 2012). Primary infection by each of the four serotypes results in long-term serotype-specific immunity and a six-month period of partial immunity to other serotypes. After this partial immunity weakens, the person becomes vulnerable to infection by the other three DENV serotypes. This secondary infection may result in the antibody-dependent enhancement/clinical manifestations (Murrell et al. 2011).

DENV-3 is one of four DENV serotypes that have five distinct genotypes (I–V). The emergence of DENV-3 strains and lineages has been increasingly reported over time (Waman et al. 2017). DENV-3 and DENV-2 serotypes are more prevalent in countries such as Thailand, Brazil, Pakistan, and India; they are also associated with a more serious clinical profile than other serotypes (de Araujo et al. 2012; Fried et al. 2010; Mehta and Shah 2018; Yousaf et al. 2018). The reemergence of DENV-3 infections occurred Brazil in 2007, in China in 2009–2010, and in Gabon in 2016–2017, which can increase the risk of repeated DENV infections in a certain area (Abe et al. 2020; Liang et al. 2013; Rodriguez-Barraquer et al. 2011).

DENV encodes three structural proteins: Capsid (C), Membrane (M) or Premembrane (prM), and Envelope (E). DENV C protein is a highly simple protein with a molecular weight of ~ 11 kDa that is involved with RNA interaction in nucleocapsid assembly. Membrane-associated protein is a ~ 26 kDa M glycoprotein that promotes the development of E protein in mature virus particles to help distinguish the immune response from different flaviviruses. The E protein is a surface protein of ~ 55 kDa, which is a large constituent of virus particles that controls binding and fusion to the host cell membrane (Cardosa et al. 2002; Ma et al. 2004; Modis et al. 2004). The expression of prM-E or co-expression with the CprME structural proteins was an effective approach for the development of recombinant flavivirus virus-like particles (VLPs). The C protein can stabilize the assembly of the VLPs, although it is not necessary for particle formation (Krol et al. 2019). VLPs are self-assembled particles, which consist of viral structural proteins. They can imitate the conformation of a genuine native virus without genomic DNA or RNA, thus making them a viable option to live-attenuated vaccines (Urakami et al. 2017).

The manufacture of dengue vaccines that involve relatively small amounts of material expressed but is an extremely different type of target antigen with significant structural differences. By contrast, monoclonal antibodies share key molecular characteristics and require a production system which delivers high-performance and high-cost-efficient systems (Legastelois et al. 2017). Compare to *E. coli*, yeast and plant expression system, expression of Dengue structural protein in silkworm expression system will be provided with more the essential lipids, molecular chaperons, and post-translational modifications that are required for the correct membrane insertion, folding, and function of eukaryotic integral membrane proteins. Those factors will improve the forming and enhance the functionalize of the VLPs (He et al. 2014; Kato et al. 2010).

Silkworms have a strong potential for application in recombinant protein processing through the production of human proteins for therapeutic use. Moreover, silkworm expression system is easy in scale up and is possible to produce mass production without any especial equipment. Several studies support the use of insect cells in manufacturing affordable antigens and recombinant vaccines. Furthermore, insect larvae, including those from silkworms, have been used for large-scale VLP development and manufacturing (Vipin Kumar Deo 2012).

In this study, we prepared dengue virus-like particles (DENV-LPs) consisting of 3CprME and 3prME polypeptides, which were expressed using *Bombyx mori* nucleopolyhedrovirus (BmNPV) bacmid in silkworms. We observed the formation of VLPs and used a mouse model to verify antibody production.

## Materials and methods

### Construction of recombinant BmNPVs

In this study, we used the coding sequences for CprME and the prME polypeptide (GenBank: KU050695, Genewiz, New Jersey, USA). The anchor region of the capsid coding sequences for CprME was deleted for increased expression level and avoiding halt viral particle formation (Nasar et al. 2020). A linker sequence (GGGGSGGGGS) and PA-tag sequence (EGGVAMPGAEDDVV) were fused in the C-terminus and amplified by polymerase chain reaction (PCR) using a template (the synthetic gene described above). A set of primers (3CprME-F and 3CprME-R-EcoRI, Table 1) was used as a template for the DENV-3CprME coding sequence. The DENV-3prME primer set (3prME-F and 3prME-R-EcoRI, Table 1) was used to isolate the DENV-3prME coding sequence. The PCR protocol was as follows: initial denaturation at 98 °C for 10 s; 35 cycles of 98 °C for 10 s, 55 °C for 5 s, and 72 °C for 20 s; 72 °C for 3 min for the final extension. A thermal cycler (TaKaRa, Kyoto, Japan) was used to carry out the PCR reaction. Each construct was ligated into pFastbac1 (Thermo Fisher Scientific K. K., Tokyo, Japan), and the resulting vector was introduced into *Escherichia coli* BmDH10bac CP^−^ Chi^−^ (Motohashi et al. 2005). The recombinant products, which included the BmNPV/3CprME and BmNPV/3prME bacmids, were extracted from white colonies, respectively. Each recombinant BmNPV bacmid was mixed with chitosan (Sigma–Aldrich, Tokyo, Japan) and injected into fifth instar silkworm larvae (Ehime Sansyu, Ehime, Japan). The hemolymph was collected from the larvae at 6–7 days post-injection (dpi) and mixed with a 1 mM solution of 1-phenyl-2-thiourea (Kato et al. 2016; Motohashi et al. 2005). The aliquots of hemolymph were kept at − 80 °C before use.

**Table 1:** Used primers

Name	5′-3′
3CprME-F	TAA TGG ATC CAT GAA TAA CCA GCG CAA GAA
3CprME-R-EcoRI	TAA TGA ATT CTC AGA CTA CGT CGT CTT CCG C
3prME-F	TAA TGG ATC CAT GTT TCA TCT CAC TTC CCG TGA TGG C
3prME-R-EcoRI	TAA TGA ATT CTC AGA CTA CGT CGT CTT CCG CAC
pFastBac1-F	TAT TCC GGA TTA TTC ATA CC
pFastBac1-R	ACA AAT GTG GTA TGG CTG ATT
pUC/M13-F	CCC AGT CAC GAC GTT GTA AAA CG
pUC/M13-R	AGC GGA TAA CAA TTT CAC ACA GG

Underlines indicate restriction enzyme cleavage sites

### Expression and purification of 3CprME and 3prME polypeptides in silkworm larvae

Fifth instar silkworm larvae (Ehime Sansyu) were injected with hemolymph that was diluted 100-fold in phosphate-buffered saline (PBS, 137 mM NaCl, 2.7 mM KCl, 8 mM Na_2_HPO_4_, and 2 mM KH_2_PO_4_, pH 7.4), and raised on an artificial diet (Silkmate S2, Nosan Co., Yokohama, Japan). The legs of the larvae were cut to collect the hemolymph. The fat bodies were collected by dissecting the larvae; 1 mL of Tris-buffered saline containing 0.1% Triton X-100 (TBST) was added to every 0.1 g of fat body and sonicated for a total of 5 min at 20-s intervals, followed by a 10-s break (Vibra Cell VC 130 PB, Sonics & Materials Inc., Newtown, USA). After sonication, the fat body suspension was centrifuged (Kubota 3700, Tokyo, Japan) for 10 min at 12,000×*g*, 4 °C. The soluble fraction of the silkworm fat body suspension was mixed with 200-µL beads tagged with anti-PA antibody (FUJIFILM Wako Pure Chemical, Osaka, Japan) at 4 °C for 2 h. The mixed beads were collected and washed five times with four times of bead volumes of TBS buffer (20 mM Tris–HCl and 150 mM NaCl). The elution was performed with a 0.1 M glycine–HCl solution (pH 3.0), and five fractions were collected to recover the PA-tagged target proteins. Amicon Ultra centrifugal filters (Merck Japan, Tokyo, Japan) were used to concentrate the evaluation by ultrafiltration. The concentrations of the eluate were measured using a BCA protein assay kit (Thermo Fisher Scientific K. K.).

The 3CprME and 3prME constructs were also expressed in Bm5 cells and silkworm pupae (Ehime Sansyu). Bm5 cells were provided by Prof. K. S. Boo (Insect Pathology Laboratory, School of Agricultural Biotechnology, Seoul National University, Seoul, South Korea). Sf-9 and Bm5 cells were maintained at 27 °C in Sf-900II serum-free medium (Thermo Fisher Scientific K.K.) supplemented with 1% fetal bovine serum (Thermo Fisher Scientific K.K.) and Antibiotic–Antimycotic solution (Thermo Fisher Scientific K.K.).

### Sodium dodecyl sulfate-polyacrylamide gel electrophoresis (SDS-PAGE) and western blot

SDS-PAGE using 10% or 12% gels was used to separate the proteins. Western blotting was subsequently performed by blotting the separated proteins onto an Immobilon-P polyvinylidene fluoride membrane (Merck Japan) using the Mini Trans-Blot Electrophoretic Transfer Cell (Bio-Rad, Hercules, CA, USA). After blotting, the membrane was blocked in 5% nonfat milk (FUJIFILM Wako Pure Chemical) in TBST (pH 7.6) and incubated in a rat anti-PA tag antibody (1:10,000; FUJIFILM Wako Pure Chemical). Other primary antibodies used included the anti-DENV E antibody (1:3000; GeneTex, Irvine, CA, USA) or the mouse anti-DENV prM antibody (1:3000; GeneTex). After incubating membranes with the primary antibodies and washing three times with TBST, the membranes were incubated for 1 h in horseradish peroxidase (HRP)-conjugated anti-rat IgG antibody (1:10,0000; FUJIFILM Wako Pure Chemical). Immobilon Western Chemiluminescent HRP substrate (Merck Japan) was used for the detection of protein bands. Membranes were imaged using a Fluor-S MAX Multi-Imager (Bio-Rad).

### Transmission electron microscopy (TEM) and immunoelectron microscopy (IEM)

TEM and IEM were carried out as previously described (Utomo et al. 2019) with minor modifications. The purified antigen sample was added to the Cu-Grid transmission electron microscope (Nisshin EM Co., Ltd., Tokyo) and incubated for 30 s at room temperature, washed with 30 μL of PBS, and incubated for 30 s. This procedure was repeated three times. For IEM, 30 μL of 2% v/v bovine serum albumin (BSA) was used for blocking after the distilled antigen sample was added, and the sample was subsequently washed three times with PBS. The Cu-Grid was washed sequentially. Samples were incubated with Dengue virus anti-envelope rabbit polyclonal antibody (1:30; FUJIFILM Wako Pure Chemical) and goat anti-rabbit IgG conjugated to gold nanoparticles (1:50; FUJIFILM Wako Pure Chemical) for the first and secondary antibodies, respectively. The Cu-Grid was treated with 2% phosphotungstic acid, and the samples were analyzed using the TEM apparatus.

### Heparin-binding assay

The heparin-binding assay was carried out as previously described (Utomo et al. 2019), with minor modifications. Biotin-labeled heparin (6 ng/mL; Sigma–Aldrich Japan) and heparin (1.8 ng) were immobilized onto avidin-coated microplate wells (blocking-less type) (Sumitomo Bakelite, Tokyo, Japan) washed three times with PBS. We used 2 µg BSA for negative control. Purified proteins (0.5, 1, 5, and 10 μg/mL) were added into wells, incubated at room temperature for 1 h, and subsequently washed with phosphate-buffered saline containing 0.1% Tween 20 (PBST). After serial washing, the rat anti-PA tag antibody (1:1000; FUJIFILM Wako Pure Chemical) and HRP-conjugated anti-rat IgG antibody (1:1000; FUJIFILM Wako Pure Chemical) were used as the primary and secondary antibodies, respectively. For detection, 100 µL of substrate (0.1 mg/mL 3.3′,5.5′-tetramethylbenzidine [TMB] in 100 mM sodium acetate [CH_3_COONa], pH 6.0) were added to each well with 0.2% (v/v) of 30% hydrogen peroxide. We added 50 μL of 1 N H_2_SO_4_ to each well to stop the reaction. Absorbance was measured at 450 nm.

### VLP antigenicity by enzyme-linked immunosorbent assay (ELISA)

Direct ELISA was used to detect an interaction between antigens, 3CprME, 3prME, the mock silkworm fat body (negative control), and sera. Two types of sera were used: mouse sera immunized with DENV tetravalent DNA vaccine (mice-Ab) (Putri D.H., personal communication, June 2017), and sera from dengue patients (human-Ab) [NS1(+), RT-PCR (+)]. The dengue patient sera were collected during a dengue community study that occurred from March 2010 until December 2011. Ethical approval was given by the Research Ethical Committee of the Faculty of Medicine, Universitas Indonesia, No. 71/PT02.FK/ETIK/2009. RT-PCR positively confirmed the human-Ab from dengue patient sera based on the Lanciotti method (Lanciotti et al. 1992). Both of the sera originated from stocks from the Department of Microbiology, Faculty of Medicine, Universitas Indonesia.

For each diluted sample, 100 µL of sample (20 ng/mL) in coating buffer (0.05 M Carbonate-bicarbonate, pH 9.6), was applied to a 96-well ELISA microplate, followed by incubation at 4 °C overnight. After incubation, the coating solution was discarded, and a 100 µL blocking solution (5% skim milk in PBS) was added into each well and incubated for 1 h at 37 °C. The plates were then washed serially with PBST, followed by the addition of 100 µL of mouse-Ab or human-Ab in PBS (1:5000). Plates were then incubated at 37 °C for 1 h and washed three times with wash buffer. Next, 100 µL of goat anti-mouse or anti-human IgG-HRP-conjugated antibody (1:5000) was added to each well. Plates were incubated at 37 °C for 1 h and washed sequentially. TMB substrate (50 µL) was applied and incubated for 10 min. We added 50 µL 1 M H_2_SO_4_ to stop the reaction. The absorbance was read at 450 nm.

### Immunization of mice

A total of 12 BALB/c mice, (4–6 weeks old) were divided into four groups: (i) negative control (immunized with PBS), (ii) immunized with 3CprME, (iii) immunized with 3prME, and (iv) immunized with Alhydrogel as an adjuvant. All mice were housed in a temperature-controlled, light-cycled room. Each mouse was immunized three times via intraperitoneal injection with 50 µg purified 3CprME and 3prME proteins with Alhydrogel adjuvant within a 2-week interval. Blood samples were collected via the tail vein after 0, 16, and 30 days. Sera were isolated and stored at − 80 °C until further analysis. All animal procedures were conducted in compliance with the established guidelines from the Animal Laboratory of Center of Pharmaceutical and Medical Technology, Agency for Technology Assessment and Application (BPPT), Indonesia. Animal experimental protocols were reviewed and approved by the Research Ethical Committee for the Faculty of Medicine, Universitas Indonesia, No. KET-476/UN2.F1/ETIK/PPM.00.02/2019.

## Results

### Expression of 3CprME and 3prME polypeptides in silkworm

**Fig. 1:**
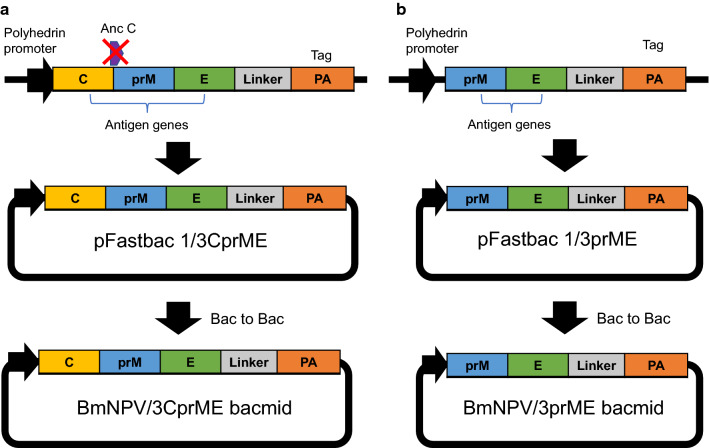
Construction of recombinant dengue virus (DENV) structural proteins expressed in this study. **a** 3CprME with the deletion of the anchor C region and **b** 3prME polypeptides of DENV-3 were expressed in silkworms, pupae, and Bm5 cells as fusion proteins with a PA-tag

**Fig. 2:**
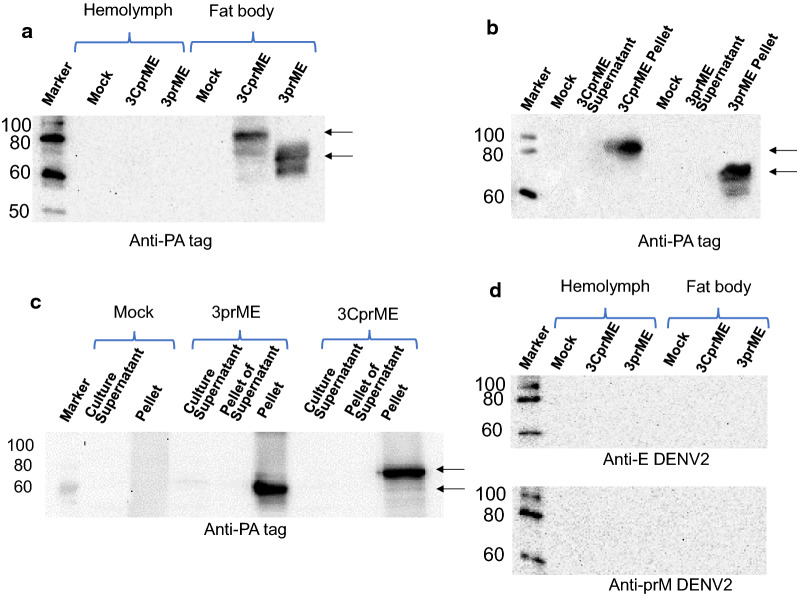
Expression of 3CprME and 3prME polypeptides in silkworm larvae (**a**), pupae (**b**), and Bm5 cells (**c**). In the case of silkworm larvae, hemolymph, and fat body were collected after infection with recombinant BmNPV. Homogenates of each sample were prepared according to the protocol described in “Materials and methods”. The 3CprME and 3prME polypeptides were detected by western blot using rat anti-PA tag as a primary antibody. **d** Hemolymph and fat body from silkworm larvae were verified for cross-reaction using mouse anti-E antibody (anti-E DENV-2) and mouse anti-prM antibody (anti-prM DENV-2) of DENV serotype 2

The DENV structural proteins consist of the C, prM, and E proteins, which are translated in the same order at the beginning of the polyprotein. The constructed BmNPV/3CprME (Fig. 1a) and /3prME (Fig. 1b) bacmids were injected into silkworm larvae, and the silkworm hemolymph and fat body were collected at 5 dpi. Expressions of 3CprME and 3prME were confirmed in fat body samples, and their molecular weights were 85 and 75 kDa, respectively. These molecular weights corresponded to the estimated protein sizes (Fig. 2a). Furthermore, both constructs were expressed in silkworm pupae (Fig. 2b) and Bm5 cells (Fig. 2c). Anti-DENV-2 prM and anti-DENV-2 E antibodies were used to analyze the cross-reactivity of these proteins to antibodies of DENV-2. The specific bands for 3CprME and 3prME were not detected using these antibodies (Fig. 2d). These results indicate no cross-reactivity between DENV-2 specific antibodies with both recombinant 3CprME and 3prME expressed in silkworms.

### Purification of 3CprME and 3prME polypeptides

**Fig. 3:**
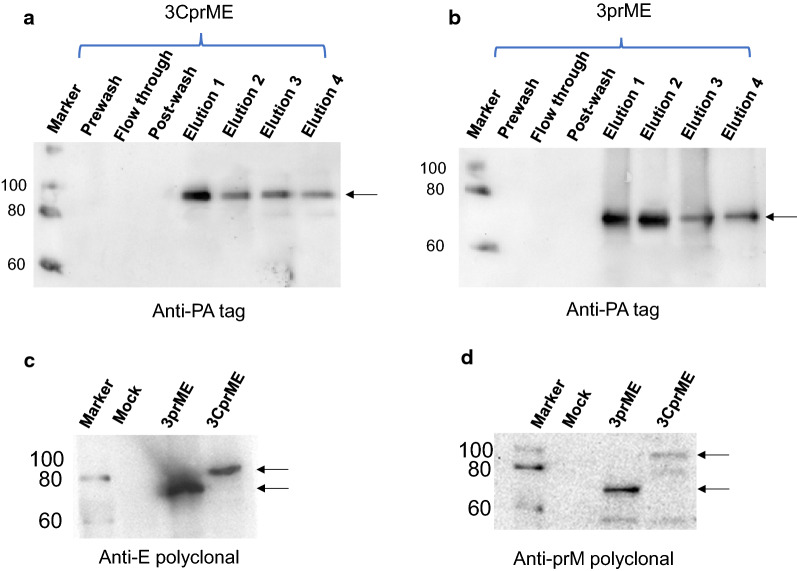
Western blot of purified 3CprME (**a**) and 3prME (**b**) polypeptides. Each protein was purified from silkworm larvae fat body using PA-tagged protein purification gel column chromatography. The E and prM were verified using rabbit anti-E protein polyclonal (**c**) and rabbit anti-prM protein polyclonal (**d**) antibodies

The successful purification of the 3CprME and 3prME polypeptides using affinity chromatography was confirmed by western blot using an anti-PA-tag antibody. The imaged blot showed a band at ~ 85 kDa corresponding to 3CprME in elution 1–4 and ~ 75 kDa for 3prME in elution 1–4 (Fig. 3a, b). To confirm whether the purified 3CprME and 3prME polypeptides contained the prM and E proteins, western blot was carried out using anti-prM or anti-E antibodies. Bands at approximately ~ 85 kDa and ~ 75 kDa were confirmed for 3CprME and 3prME, respectively (Fig. 3c, d). These results confirm that the purified 3CprME and 3prME polypeptides contained the prM and E proteins. The purified yield of 3CprME and 3prME was 420 μg/10 silkworms and 380 μg/10 silkworms, respectively. To compare the expression level, Bm5 cells, larva and pupa were compared (Additional file 1: Figure S1). Based on Additional file 1: Figure S1 result and purified result of larvae, a larva and a pupa assume to produce 42 µg and 151 µg of the purified 3CprME, respectively. These results correspond to 4 mL and 15 mL of Bm5 cell culture, respectively.

### Morphology of the 3CprME and 3prME polypeptides

**Fig. 4:**
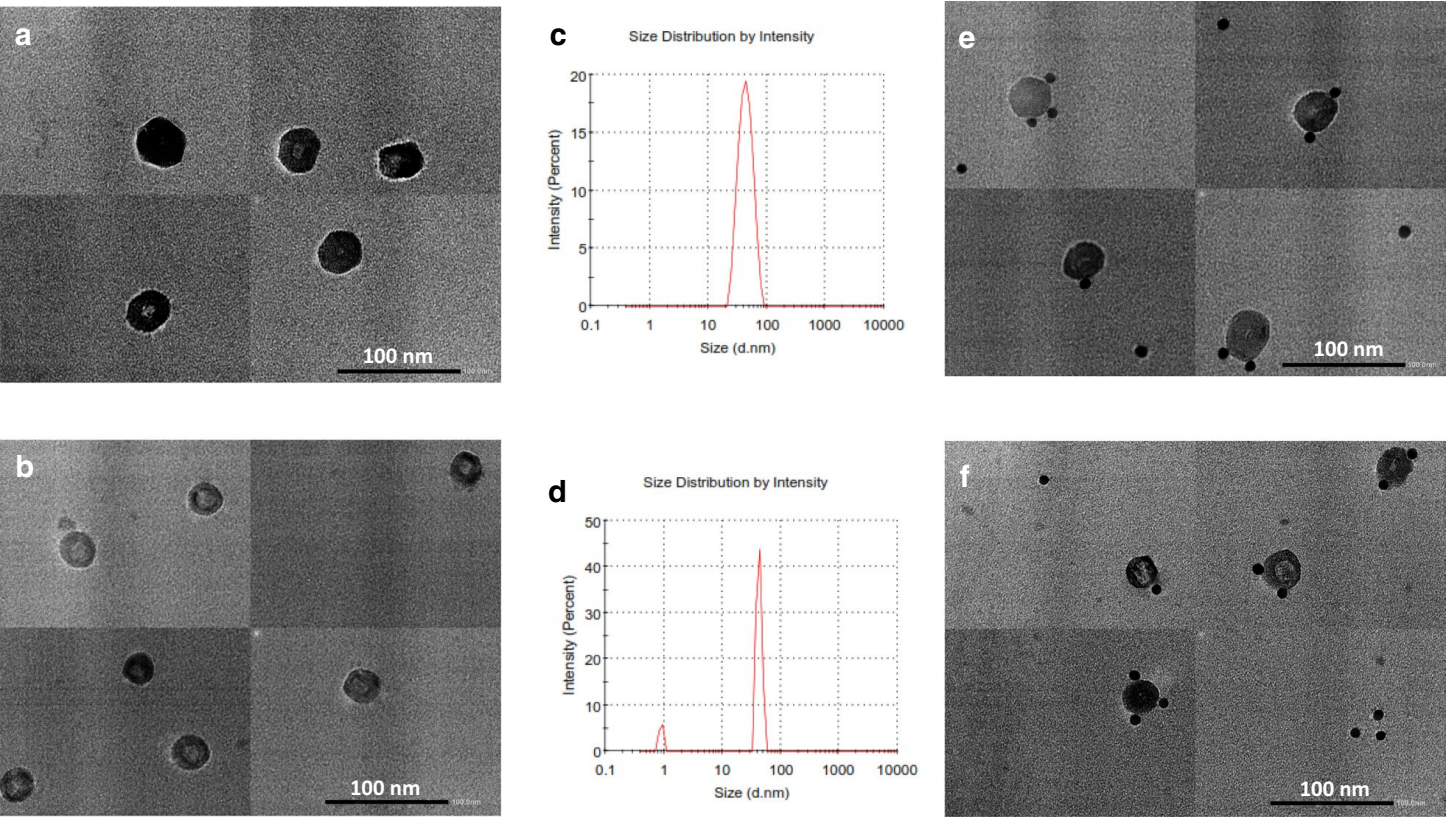
TEM analysis of purified 3CprME (**a**) and 3prME (**b**) polypeptides. The purified 3CprME (**c**) and 3prME (**d**) polypeptides were immunogold-labeled using an anti-E polyclonal antibody and analyzed via IEM. Black spots in **c** and **d** indicate immunogold particles. Dynamic light scattering (DLS) was used to analyze the size distribution for 3CprME (**e**) and 3prME (**f**) polypeptides

TEM and IEM were performed to confirm the morphology of the 3CprME and 3prME polypeptides. Spherical structures with a size of 30–55 nm were observed (Fig. 4a, b) and it is strengthened by the data for digital light scattering (Fig. 4c, d). The IEM images revealed gold nanoparticles bonded to the surface of a lipid bilayer of spherical structured VLPs (Fig. 4e, f). The binding of gold nanoparticles on the surface of the spherical structures indicates the presence of E protein. These results suggest that 3CprME and 3prME polypeptides expressed in silkworm generate DENV-3 VLPs (DENV-LP/3CprME and /3prME).

### Heparin-binding assay of the DENV-LPs/3CprME and /3prME

**Fig. 5:**
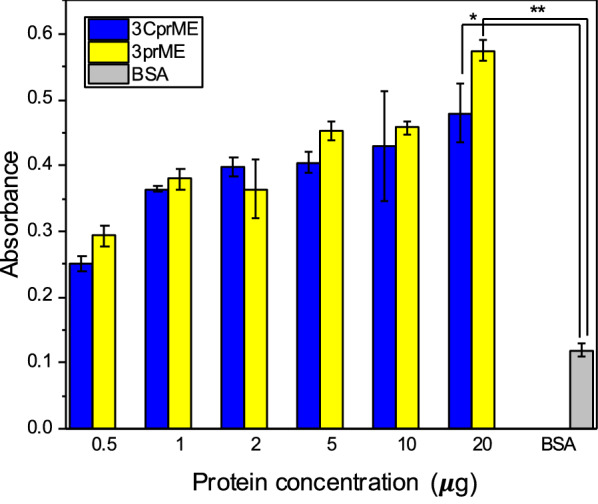
Binding assay of DENV-LPs/3CprME and /3prME to heparin using ELISA. Heparin (1.8 ng) was coated onto each well of a microplate and the ELISA protocol was carried out according to “Materials and methods” section

The heparin-binding assay was performed to confirm the expression of E protein domain III (EDIII) on the surface of the DENV-LPs. The binding assay for the purified 3CprME and 3prME polypeptides was performed using the heparin-immobilized microtiter plates. There was a positive relationship between absorbance and the quantity of E protein compared with BSA (Fig. 5). These results suggest that the 3CprME and 3prME contain the EDIII on the surface of their DENV-LPs.

### Antigenicity of DENV-LPs/3CprME and /3prME

**Fig. 6:**
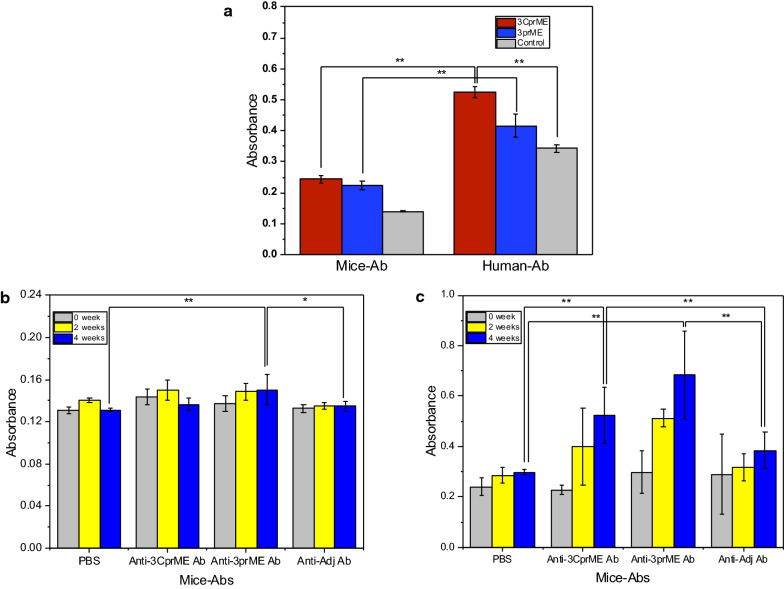
**a** The interactions between DENV-LPs/3CprME and /3prME with sera from mice immunized with the DENV tetravalent DNA vaccine and mixed sera from dengue patients [NS1(+), RT-PCR (+)] were investigated. Binding reactions were analyzed using direct ELISA as described (Welch t-test, **p < 0.01). Specific IgG generation by DENV-LPs/3CprME (**b**) and /3prME (**c**). BALB/c mice were intraperitoneally immunized with 50 μg monovalent DENV-LPs/3CprME and/3prME. At 2 and 4 weeks, sera were collected and analyzed for specific IgGs (Welch t-test, *p < 0.05, **p < 0.01)

The antigenicity of purified DENV-LPs/3CprME and /3prME were characterized by direct ELISA. Our results showed that 3CprME and 3prME showed greater reactivity to mouse-Ab and human-Ab compared with the negative control (Fig. 6a). This result indicates that DENV-LPs/3CprME and /3prME may be used to discriminate against sera from DENV-3-infected humans.

### DENV-LPs/3CprME and /3prME elicited viral-specific IgG

BALB/c mice were immunized intraperitoneally three times at three-week intervals with 50 μg of DENV-LPs/3CprME and /3prME with an Alhydrogel adjuvant. The antibody titers were measured in mouse sera by ELISA at two weeks after the last immunization. Immunization with DENV-LPs/3CprME and /3prME induced the generation of anti-3CprME and anti-3prME Abs, which were analyzed for specific binding using their own antigens and reciprocal. Both specific antibodies showed a low affinity for the DENV-LPs/3CprME (Fig. 6b). On the contrary, the anti-3CprME Abs and anti-3prME Abs recognize the DENV-LPs/3prME (Fig. 6c). These results indicate that the DENV-LPs/3prME is suitable to elicit the generation of specific antibodies.

## Discussion

Our previous study showed that the DENV-LP serotype 2 was expressed in the hemolymph of silkworm larvae (Utomo et al. 2019). In this study, the DENV-3 structural proteins 3CprME were expressed by removing the anchor sequence of capsid protein between the C and prM polypeptides. The purpose of the anchor capsid deletion was to increase protein expression; however, the protein cannot be released into the hemolymph of the silkworm without the anchor sequence. The expressed 3CprME and 3prME polypeptides were detected in the fat body soluble fraction, not the hemolymph.

Recombinant flaviviral VLPs can be efficiently generated with or without the C protein by expressing proteins prM and E (Sangiambut et al. 2013). The absence of prM and/or C did not affect the self-assembly of the E in VLP formation (Chang et al. 2001; Mani et al. 2013; Putnak et al. 2003). As indicated by heparin-binding assay results, EDIII was present on the surface of the VLPs because heparin in the cell membrane is known to interact with EDIII, the putative receptor-binding domain in the flavivirus E protein crystal structure. EDIII also contains epitopes that block viral adsorption and is targeted by many antibodies, including serotype-specific neutralizing monoclonal antibodies (Chen et al. 2016; Frei et al. 2018; Han et al. 2018; Hidari et al. 2013; Yang et al. 2016). With the EDIII present on the surface of the VLPs, the mediated antibody is expected to have increased viral neutralization activity, which may allow it to reduce the viral load in normal DENV infection. A neutralization assay is recommended for further study.

Both 3CprME and 3prME also showed reactivity to the mouse-Ab and human-Ab; however, the human-Ab showed a higher affinity in the direct ELISA than the mouse-Ab. Since the mouse-Ab was obtained from mice that were immunized with the tetravalent DNA vaccine, it had lower immunogenicity compared with the human-Ab that was isolated from DENV-infected patients. Mixed sera of dengue patients can react to many non-specific DENV epitopes. The DENV tetravalent DNA vaccine is well tolerated and capable of generating a sufficient anti-dengue interferon-gamma-mediated T-cell response (Danko et al. 2018).

The DENV-LPs/3CprME and /3prME were capable of generating IgG antibodies, but the addition of the Alhydrogel adjuvant did not yield any specific binding. The detected IgG total responses in mice immunized with the 3prME showed a better profile than those immunized with 3CprME. These differences in the response to the antigens might be caused by the deletion of the anchor domain in the C region, which may lower its antibody response, even though the capsid protein is not exposed on the viral surface. Each DENV serotype carries the conserved antibody epitope that is incorporated into the N- and C-terminal regions of the C protein, which is efficiently recognized by dengue patients previously exposed to primary and secondary infections from other serotypes. The C-protein central region has an epitope of the peptide, which is primarily targeted by serotype-specific antibodies (Alves et al. 2016; Nadugala et al. 2017; Rana et al. 2018). Further analysis of the antibody response to other serotypes of the structural proteins and the different DENV serotypes are recommended.

## Supplementary information

**Additional file 1: Figure S1.** Protein expression yield of 3CprME and 3prME polypeptides in BM5 cells, silkworm larvae, silkworm pupae.

## Data Availability

All the data and materials have been provided in the main manuscript.

## Abbreviations

Ab: Antibody
C: Capsid protein
DENV: Dengue virus
DLS: Digital light scattering
E: Envelope protein
ELISA: Enzyme-linked immunosorbent assay
EDIII: Envelope protein domain III
HRP: Horseradish peroxidase
IEM: Immunoelectron microscopy
M: Membrane protein
PBS: Phosphate-buffered saline
PBST: Phosphate-buffered saline containing 0.1% Tween 20
PCR: Polymerase chain reaction
prM: Premembrane protein
TBS: Tris-buffered saline
TBST: Tris-buffered saline containing 0.1% Triton X-100
TEM: Transmission electron microscopy
TMB: 3.3′,5.5′-Tetramethylbenzidine
VLP: Viral-like particle
WHO: World Health Organization
