# Supplementary material for: Production of dengue virus-like particles serotype-3 in silkworm larvae and their ability to elicit a humoral immune response in mice
Source: AMB Express. 2020 Aug 17;10:147. doi: 10.1186/s13568-020-01087-3 (PMC7431508; doi:10.1186/s13568-020-01087-3)
Supplement: Supplementary file 1 — Additional file 1: Figure S1. Protein expression yield of 3CprME and 3prME polypeptides in BM5 cells, silkworm larvae, silkworm pupae. [file 13568_2020_1087_MOESM1_ESM.docx]

**Additional Information**

**Production of dengue virus-like particles serotype-3 in silkworm larvae and their ability to elicit a humoral immune response in mice**

**Doddy Irawan Setyo Utomo^1^, Sabar Pambudi^2^, Fithriyah Sjatha^3^, Tatsuya Kato^1,4^, Enoch Y Park^1,4,*^**

^1^ Laboratory of Biotechnology, Department of Bioscience, Graduate School of Science and Technology, Shizuoka University, 836 Ohya, Suruga-ku, Shizuoka 422-8529, Japan

^2^ Center of Pharmaceutical and Medical Technology, Agency for the Assessment and Application of Technology, Jl. Kawasan Puspiptek, Gedung I LAPTIAB, Kota Tangerang Selatan, Banten 15314, Indonesia

^3^ Department of Microbiology, Faculty of Medicine, Universitas Indonesia, Jl. Pegangsaan Timur 16, Cikini, Jakarta 10320, Indonesia

^4^ Laboratory of Biotechnology, Research Institute of Green Science and Technology, Shizuoka University, 836 Ohya, Suruga-ku, Shizuoka 422-8529, Japan

Enoch Y. Park; [park.enoch@shizuoka.ac.jp](mailto:park.enoch@shizuoka.ac.jp)

E-mails:

[doddy.irawan.setyo.utomo.16@shizuoka.ac.jp](mailto:doddy.irawan.setyo.utomo.16@shizuoka.ac.jp) (DISU)

[sabar.pambudi@bppt.go.id](mailto:sabar.pambudi@bppt.go.id) (SP)

[fithriyah31@ui.ic.id](mailto:fithriyah31@ui.ic.id) (FS)

[kato.tatsuya@shizuoka.ac.jp](mailto:kato.tatsuya@shizuoka.ac.jp) (TK)

[park.enoch@shizuoka.ac.jp](mailto:park.enoch@shizuoka.ac.jp) (EYP)

**Fig. S1** Protein expression yield of 3CprME and 3prME polypeptides in BM5 cells, silkworm larvae, silkworm pupae. The 3CprME and 3prME polypeptides were detected by western blot using rat anti-PA tag as a primary antibody. The band intensities were quantified using ImageJ (National Institutes of Health, Bethesda, USA).

To quantify the yield of protein expression, the 3CprME, and 3prME constructs were expressed in 2 mL Bm5 cells culture, one larva of the silkworm, and one pupa of the silkworm. Protein samples were using SDS-PAGE, followed by immunoblotting. 3CprME and 3prME were detected using rat anti-PA tag antibody as the primary antibody and horseradish peroxidase (HRP)-conjugated anti-rat IgG antibody as the secondary antibody, as described in the ‘Materials and Methods’ section. As shown in Fig. S1, the protein expression in BM5 cells was ~0.5 times lower, and in silkworm pupae, ~3.5 times higher, compared to expression in silkworm larvae. As mentioned in the Results section, the yield of purified protein from 10 silkworm larvae was 420 μg for 3CprME and 380 μg for 3prME. Based on Fig. S1 result and purified result of larvae, a larva and a pupa assume to produce 42 μg and 151 μg of the purified 3CprME, respectively. These results correspond to 4 ml and 15 ml of Bm5 cell culture, respectively.
